# Understanding the patient voice in gout: a quantitative study conducted in Europe

**DOI:** 10.3399/bjgpopen20X101003

**Published:** 2020-02-19

**Authors:** Marc De Meulemeester, Elsa Mateus, Hilda Wieberneit-Tolman, Neil Betteridge, Lucy Ireland, Gudula Petersen, Nina Jeanette Maske, Tim L Jansen, Fernando Perez-Ruiz

**Affiliations:** 1 GP Private Practice, Cabinet Médical Demeulemeester, Gozée, Belgium; 2 President of the Board of Portuguese League Against Rheumatic Diseases, Liga Portuguesa Contra as Doenças Reumáticas, Lisboa, Portugal; 3 Executive Director of PijnPlatform Nederland, Pijn Platform Nederland, Leiden, The Netherlands; 4 Director, Neil Betteridge Associates, London, UK; 5 Health Partner, Hall & Partners, London, UK; 6 Director Governmental Affairs & Patient Centricity, Grünenthal GmbH, Aachen, Germany; 7 Senior Manager Market Research & Insights, Department Analytics & Insights, Grünenthal GmbH, Aachen, Germany; 8 Medical Leader, Department of Rheumatology, Expert Centre Complex Gout, VieCuri Medical Centre, Venlo, The Netherlands; 9 Associate Professor, Department of Medicine, Medicine and Nursery School, University of the Basque Country, Cruces, Spain

**Keywords:** gout, uric acid, urate, quality of life, general practice, patient care, surveys and questionnaires, pain

## Abstract

**Background:**

Although commonly diagnosed, gout often remains a poorly managed disease. This is partially due to a lack of awareness of the long-term effect of gout among patients and healthcare professionals.

**Aim:**

To understand unmet needs for patients and provide insight into achieving better treatment.

**Design & setting:**

A quantitative online questionnaire collected from 1100 people with gout from 14 countries within Europe.

**Method:**

Patients were recruited to complete an online survey via healthcare professional (HCP) referral, patient associations, or market research panels. Patients were included if they had been diagnosed with gout by a physician. Prior to commencement, patients were made aware that this study was sponsored by Grünenthal. The responses collected were collated and analyses were performed.

**Results:**

Patients had an average of 2.9 gout flares within a 12-month period. Although 79% of patients were satisfied with treatment, inadequate gout control was also reported by 71% of patients. Furthermore, 84% experienced moderate-to-severe pain with their most recent flare. Of those who acknowledged treatment dissatisfaction, only 24% discussed other options with their GP. Most patients reported irregular follow-up and serum uric acid (sUA) monitoring. In addition, loss of belief that more can be done was a key barrier for patients.

**Conclusion:**

Patients reported severe pain and social burden, coupled with low treatment expectation and lack of awareness of target sUA. Education around knowing and reaching sUA target is needed so that patients can receive and GPs can deliver higher quality management.

## How this fits in

Although common, gout remains a poorly managed condition, partially due to a lack of awareness of treatment options, patient self-blame, and poor knowledge of gout pathophysiology. This study demonstrates that most patients with gout who participated in the survey experienced inadequate gout control, as evidenced by frequent flares associated with debilitating moderate-to-severe pain. Most patients reported irregular follow-up and disease monitoring with their physician, unproductive discussions about treatment options, and a loss of belief that more could be done to treat their condition. This study therefore illustrates the need for both physician and patient education, in line with European League Against Rheumatism (EULAR) recommendations, around regular sUA monitoring and more informed discussions about treatment options to improve quality of care in gout.

## Introduction

Gout is a potentially debilitating and common genetic metabolic disease.^1–3^ If not well treated, this chronic disease has a similar potential for functional and social impairment as other inflammatory arthritic conditions, including rheumatoid arthritis.^2^ Prevalence of gout is estimated to be between 0.1% and 10% worldwide.^3,4^

Despite its rising prevalence, gout is a generally misinterpreted condition.^1^ Even when appropriately diagnosed, gout often remains undertreated.^1^ This is likely due to attention given by physicians predominantly to treating only the acute component of gout. The chronic metabolic nature of gout is poorly addressed by acute treatment, as patients are generally advised to manage flares through lifestyle modifications prior to pharmacological management.^2,5^ Therefore, the potential seriousness of gout is also misunderstood by patients due to an absence of education on the underlying pathophysiology of the disease.^2^ A primary reason that gout is misunderstood may be due to a previous period defined by limited research and a relatively quiescent treatment landscape. Since the introduction of allopurinol, few new treatments have entered clinical practice. It is likely that the scarcity of research has contributed to a lack of awareness of the disease.

Patient empowerment is currently prioritised in medicine, particularly in the gout treatment guidelines.^6^ However, little information is available about current management trends and the satisfaction of patients living with gout. Furthermore, insights into how patient interactions with physicians might dictate the course of their treatment are currently sparse. To that end, quantitative research was carried out to explore unmet needs in gout from the patients’ perspective. This included questions probing the impact of gout on patients’ emotional and social wellbeing, patient experience with diagnosis, and their treatment expectations post-diagnosis. This study aims to provide insight into achieving better treatment satisfaction for both patients and physicians.

## Method

Data for this study, comprising quantitative online questionnaires, were collected from individuals with gout (‘patients’) from 14 countries within Europe: Austria, Belgium, Denmark, France, Germany, Ireland, Italy, Malta, The Netherlands, Norway, Portugal, Spain, Sweden, and Switzerland. The questionnaire was developed by Hall & Partners, an independent market research agency, on behalf of Grünenthal. Prior to initiation of the survey, the participants were made aware that this study was sponsored by Grünenthal and they gave their explicit consent to participate.

Between 13 June and 30 September 2018, patients completed a 15-minute online survey, which was available in multiple languages. Survey questions were written with the help of clinical experts and patient organisations. Patients were recruited from a mixture of sources, including via physician referral, patient associations, and market research panels (Dynata [formerly Research Now SSI] and Toluna), and were eligible for inclusion if they had been diagnosed with gout.

The survey collected information on the frequency and duration of flares. As such, gout was considered uncontrolled if patients had experienced ≥1 flare in the past 12 months. Flare frequency was used as a surrogate for sUA because awareness of sUA is low, and it is not routinely measured. Diagnosis of flare was patient-reported as it was the patient who completed the survey. Median time since diagnosis and proportion of patients on urate-lowering therapy (ULT) was also collected to ensure that patients had appropriate time to be offered ULT as part of their gout management.

Moderate-to-severe pain was defined by a range of 0–10 using an 11-point visual analogue scale. Based on the pain experienced during the worst flare, mild pain was defined as a rating from 0–4, moderate as 5–7, and severe as 8–10. Information on comorbidities was also collected, as defined and identified by patient responders.

The responses collected in the online questionnaires were collated by the research team at Hall & Partners using Askia ANALYSE (version 5.3.5.5). Answers were analysed using *t*-tests between subgroups, automatically carried out by the software. The patients’ answers were analysed by the number of gout flares reported in the last 12 months prior to the survey.

## Results

### Patient demographics

Study inclusion criteria were met by 1100 adult gout patients across Europe (Table 1). Most patients (78%) were male, with a mean age of 55 years (range 18–65). At the time of the survey, 56% of patients were employed, studying, or searching for employment. Gout was moderate in severity, with an average of 2.9 flares experienced in the last 12-month period. Just over half of all patients were on ULT (58%), and the majority (77%) had ≥1 comorbidities. The most common comorbidity was high blood pressure, followed by high cholesterol and obesity.

**Table 1.** Patient demographics including comorbidities (*n* = 1100)

**Patient demographics**	*n* (%)
Male	858 (78)
Mean age, years (range)	55 (18–65)
Mean age at diagnosis, years (range)	45 (23–85)
Mean number of flares within a 12-month period (range)	2.9 (0–12)
Length of most recent flare, days (range)	5.1 (1–16)
Average time since most recent flare, months (range)	4.5 (1–12)
Receiving ULT	638 (58)
Employed, studying or searching for employment	616 (56)
**Comorbidities**	847 (77)
Chronic kidney disease	187 (17)
Type 2 diabetes	253 (23)
Overweight/obesity	440 (40)
Hypercholesterolaemia	451 (41)
Hypertension	583 (53)

ULT = urate-lowering therapy.

The survey explored three themes: impact of gout on patients; treatment satisfaction; and gout management. The following sections present research findings across those three themes.

### Impact of gout on patients

As revealed by this study, 71% (*n* = 779/1100) of patients had uncontrolled gout. Of those, 44% (*n* = 346) experienced ≥3 flares in the last 12 months. Although uncontrolled gout was defined as ≥1 flare in the last 12 months, data presented herein focuses on patients who have had >5 flares as they had the most extreme need for improved care.

In addition to gout flares, gout was highly associated with pain: 84% (*n* = 924/1100) of patients reported experiencing moderate-to-severe pain with their most recent flare and 93% (*n* = 1020/1100) claimed that pain intensity was severe (8–10) in their worst flare. When describing gout, 34% (*n* = 374/1100) responded that the pain was so intense it was unbearable. Patients described their gout as inconvenient, agonising, and frustrating.

**Figure 1.**
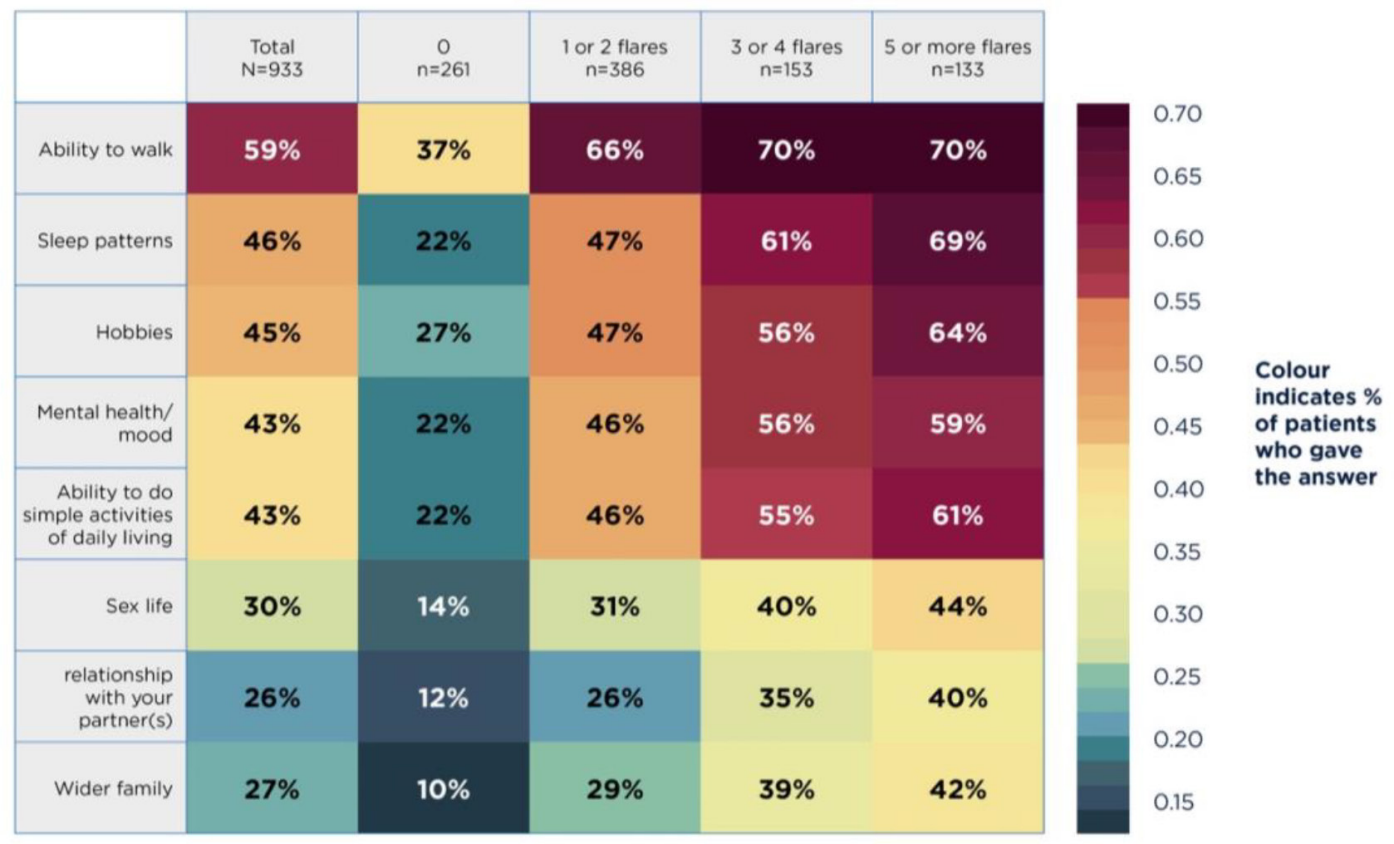
Impact of gout on patient activities versus level of control (*N* = 933 due to missing data)

Gout also affected patients’ physical and emotional function. As shown in Figure 1, 59% (*n* = 550/933) of patients said that gout impacted their ability to walk, 43% (*n* = 401/933) reported changes in mood and mental health, and 26% (*n* = 243/933) reported difficulties in their relationship with their partner.

Poor gout control, in terms of flare frequency, was particularly an issue for younger patients: 93% (*n* = 128/138) of patients aged 18–35 years had ≥1 flare in the last 12 months versus 63% (*n* = 365/579) of patients aged ≥56 years. Additionally, 25% (*n*= 35/138) of younger patients reported that they or a family member had retired or lost their job because of their gout, and 40% (*n* = 55/138) missed ≥5 days of work due to symptoms in the last 12 months. In terms of emotional impact, 44% (*n* = 61/138) of younger patients had gout negatively impact their personal relationships and 41% (*n* = 57/138) reported a negative impact on sexual relationships. In contrast, 19% (*n* = 110/579) and 23% (*n* = 133/579) of older patients said their gout impacted personal relationships and sexual relationships, respectively.

### Treatment satisfaction

**Figure 2.**
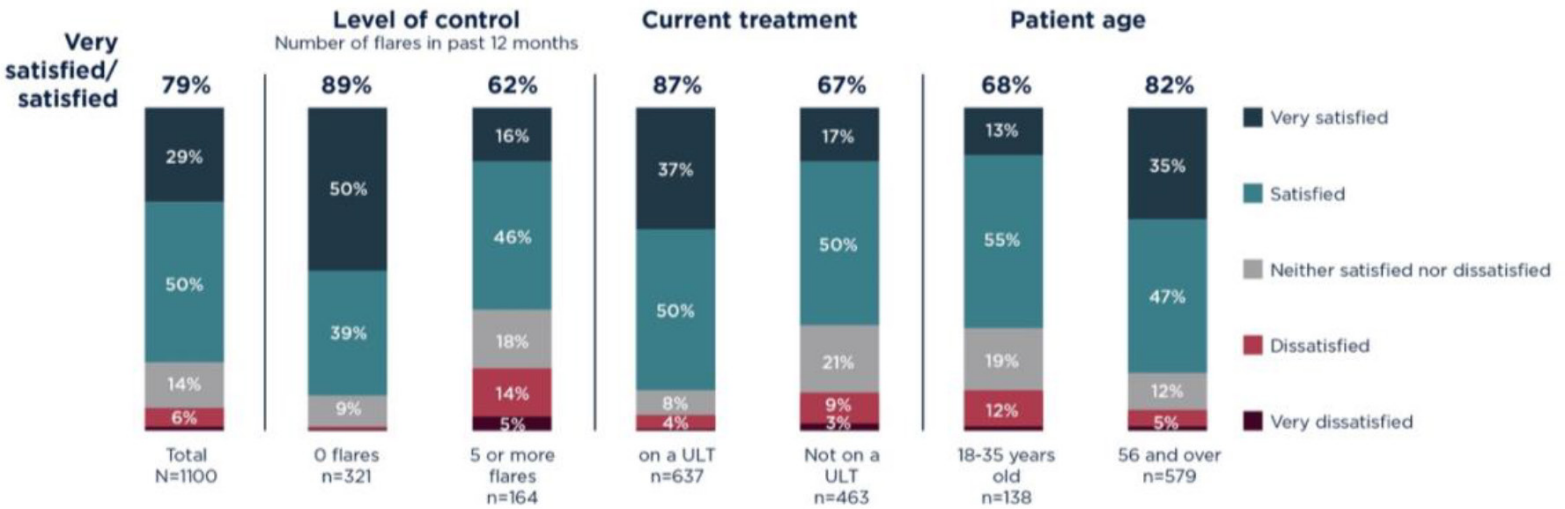
Treatment satisfaction versus level of control, treatment, and age

In total, 79% (*n* = 865) of patients claimed to be satisfied with their current treatment. Of those who acknowledged treatment dissatisfaction, only 24% (*n* = 56/235) discussed other options with their GP. Satisfaction decreased as number of flares experienced increased, yet still 62% (*n* = 102) of patients experiencing ≥5 flares in the last 12 months reported satisfaction. When stratified by treatment type, those receiving ULT said that they were more satisfied than those receiving colchicine, pain killers or corticosteroids, a non-ULT treatment, or no treatment at all (87%, *n* = 554/637 vs 67%, *n* = 310/463). Furthermore, overall satisfaction was higher in older patients compared with younger patients (82%, *n* = 475/579 vs 68%, *n* = 94/138), irrespective of treatment type (Figure 2).

Treatment satisfaction did not equate to gout control as defined by reported flare frequency. Of the 865 patients (79%) who said that they were satisfied with the current medicines that their physician had prescribed or recommended, 67% (*n* = 579) had ≥1 flare while 27% (*n* = 233) had ≥3 flares in the last 12 months.

Of the 6% (*n* = 66) dissatisfied with their treatment, only 36% (*n* = 24) discussed treatment changes with their physician while 22% (*n* = 15) had no treatment discussion after mentioning dissatisfaction. Furthermore, 20% (*n* = 13) of patients dissatisfied with their treatment raised no management concerns at all with their physician.

### Gout management

#### Diagnosis

In this survey, it was reported that GPs were involved in diagnosis of the disease (73%, *n* = 803), discussion of gout (68%, *n* = 748), and treatment of last gout flare (59%, *n* = 649). Conversely, rheumatologists were less involved at all touchpoints: diagnosis (8%, *n* = 88), discussion (15%, *n* = 165), and treatment (11%, *n* = 121) of last gout flare.

Although 52% (*n* = 572) of patients were diagnosed during or after their first flare, 22% (*n* = 242) said that they were not diagnosed until they had experienced ≥4 flares. This was particularly an issue with younger patients, where 35% (*n* = 38/109) of patients aged <25 years were not diagnosed until they had experienced ≥4 flares in contrast to 19% (*n* = 85/449) of patients aged 36–55 years and 11% (*n* = 27/249)of patients aged ≥56 years. This is evidenced by the fact that time-to-diagnosis was associated with flare frequency in patients included in this survey: 39% (*n* = 184/473) of patients diagnosed during or after their first flare reported ≥3 flares in the last 12 months compared with 45% (*n* = 88/196) of patients diagnosed on or after their fourth flare.

#### Monitoring

After a diagnosis of gout was made, most patients did not have regular follow-up appointments and were not monitored for sUA. Across Europe, higher frequency follow-ups were focused on the patients with the greatest need. For instance, 76% (*n* = 124/164) of patients with ≥5 flares had follow-up visits versus 32% (*n* = 103/321) of those who had not flared in the last 12 months. Higher follow-up frequency was not only associated with flare frequency but also with patient age: 53% (*n* = 73/138) of patients aged 18–35 years reported ≥2 visits within the last 12 months versus 38% (*n* = 220/579) of patients aged ≥56 years. Similarly, frequency of sUA monitoring was low: 53% (*n* = 583/1100) of patients’ sUA levels were measured <2 times per year.

Sixty per cent (*n* = 660) of patients said that they had very little or no knowledge about their condition. Half of patients (50%, n = 82/164) suffering ≥5 flares in the last 12 months reported knowing only basic facts about their disease. Even in areas that patients claimed to have the most familiarity with, such as sUA levels, knowledge appeared to be superficial: although 86% (*n* = 946/1100) of patients knew that high sUA levels could lead to future flares, only 32% (*n* = 352/1100) undergoing sUA testing were aware of their sUA target.

#### Treatment

Among patients surveyed, 49% (*n* = 539) said that they wanted more consultation time to ask questions and discuss treatment options. In addition to the desire for more time, loss of belief that more could be done was a key barrier to gout discussions for patients with poorer gout control (≥5 flares per year). For instance, 42% (*n* = 462) of patients told their physician about less than half of the flares they experienced over the last year.

Once diagnosed with gout, 58% (*n* = 638) of patients were prescribed ULT. ULT use was higher in older patients: 62% (*n* = 359/579) of patients aged ≥56 years versus 49% (*n* = 68/138)of patients aged 18–35 years. Most patients received ULT in combination with other medication: 43% (*n* = 473/1100) received pain killers, 25% (n = 275/1100) received colchicine, and 12% (*n* = 132/1100) received corticosteroids. Of those patients not receiving ULT, 38% (*n* = 16/463) reported ≥3 flares in the last 12 months and 81% (*n* = 375/463) have had ≥1 flares in the same time.

Fifty-seven per cent (*n* = 627) of patients reported that they felt resigned to the fact their current treatment was the best it can get. Only 28% (*n* = 308) stated that they were interested in looking for new treatment options. Over 70% (*n* = 792/1100) of patients expressed hope that better treatments would become available, while almost 40% (*n* = 418/1100) agreed that there was a need for greater awareness among the public about the severity and impact of gout.

## Discussion

### Summary

This study included 1100 patients with gout, with a male-to-female ratio of 3:1 and a mean age of 55 years. The most common comorbidities were hypertension, hyperlipidaemia, diabetes, and obesity. About 60% of patients received ULT. This study population is similar to the InGef German healthcare database that included 62 425 gout claims.^4^ In that database, the majority of patients were male and 47% of patients were aged <65 years; the pattern of comorbidities matched this survey, and about 70% of patients received ULT.^4^ The present survey expands on the epidemiological data gathered by InGef, and provides patient and GP insights into the management of gout.

**Figure 3.**
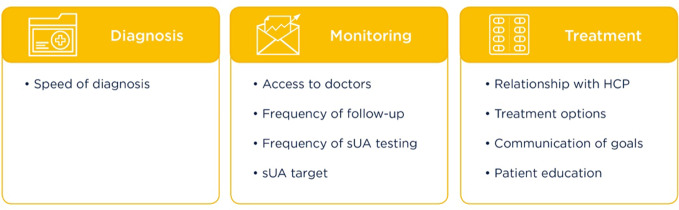
Clear areas of unmet need. HCP = healthcare practitioner. sUA = serum uric acid.

For many patients, there are clear areas of unmet need throughout the course of their disease. As detailed in Figure 3, which is a visual summary of the results presented herein, these include speed of diagnosis, frequency of follow-up and monitoring, and clear communication around treatment expectation.

The results of the survey demonstrate that a typical GP-treated gout patient in Europe is diagnosed late, and their condition may not be well controlled nor regularly monitored. This is in line with the 2016 EULAR recommendations, which highlight that gout management is still not optimal in a large proportion of patients.^6,7^ As lack of monitoring in primary care is a large barrier to treatment, regular sUA measurements are recommended to help monitor the disease, with therapy adjusted to treat-to-target to ensure optimal outcomes.^6,8^

There is a general lack of education and awareness from both patients and GPs regarding gout management. Although gout is treatable, absence of education leads to underdiagnosis and undertreatment of the condition, especially for patients who do not fit the ‘stereotypical gout’ profile (for example, younger patients). The high frequency of flares despite treatment reported in this study indicates that patients may not be receiving proper care. This reinforces the idea that some patients feel self-stigma and, as a consequence, are hesitant to discuss their condition as they feel that flares occur as a result of their own actions or inactions.^5,9^

The survey results also suggest that GPs may not recognise optimal gout control, and may not appreciate the pain and impact of gout on their patients’ quality of life.^10^ Lack of gout control, and pain management especially, can be a physical, mental, and emotional drain for patients as they and their treating GPs may feel that their gout is linked to unhealthy dietary habits. Recent research has begun to challenge that perception, showing that genetic variants have a much greater contribution to hyperuricaemia in the general population than dietary exposure.^5^ As a result, patients are not optimally managed and this mismanagement may have repercussions, such as joint damage and development of comorbidities.^9,11^ Better quality conversations are needed: patients need to be more willing to tell their GPs about the depth of their suffering and be made aware that better management can deliver better outcomes.

For younger patients, gout management can be complex. Younger patients have a longer time to diagnosis than older patients, which could have implications on their future health. Occurrence of ≥1 episode of peripheral joint or bursal swelling, pain or tenderness is sufficient for a diagnosis or classification of gout.^12^ As revealed in this survey and supported by clinical data, late diagnosis and a lack of treatment can lead to increased frequency and duration of flares.^13^ Furthermore, treatment satisfaction may also be lower in younger patients, likely due to the greater impact of gout on their social and emotional wellbeing.

For all patients, education about proper treatment is crucial to achieve better gout control.^14^ This may potentially be achieved through public health awareness initiatives, information from patients’ physicians, and with the help of patient advocacy groups. This survey revealed that over half of all patients felt resigned to the fact their current treatment was the best they could obtain. Particularly in older patients, loss of belief that more can be done is a key barrier to gout discussions, but this attitude was also observed in younger patients. There is, therefore, an opportunity to educate both patients and the public about gout management and current treatment options. For instance, sUA monitoring and reaching target sUA with ULT will facilitate proactive management of flares by patients and foster better discussions with their physicians at consultation.^15^

Similarly, physicians may not be fully aware of how poorly their patient’s gout is controlled, and this reinforces the importance of frequent sUA tests. As a result, sUA should be routinely monitored and used to direct treatment.^6,16,17^ This is supported by a recent utility analysis that suggested that monitoring twice per year after achieving target sUA was the most cost-effective approach to gout management compared with either no testing or annual testing, as assessed by flare rate and health-related quality of life modelled over a lifetime horizon.^18^

### Strengths and limitations

Few studies have been conducted within Europe to better understand the patient experience with gout.^4^ The findings presented herein represent multiple patient subgroups within the predominantly GP-treated sample (such as, age, sex, and comorbidities). However, the sampling frame of referrals, patient associations, and market research panels may limit study findings as gout patients treated by rheumatologists may already achieve better outcomes. This population of patients was not included in the current study.

A limitation of this study is the unknown impact comorbidities may have had on gout treatment satisfaction as 77% (*n* = 847) of the responders had ≥1 condition concurrent with gout. As the survey was conducted across Europe, the potential for under-representation from certain countries is also possible. Lastly, public health awareness across countries may be varied, which may confound how certain groups of patients responded to the questionnaire.

### Comparison with existing literature

In the current study, 79% (*n* = 865) of patients claimed to be satisfied with their current treatment despite the presence of recurrent flares. This is similar to a 2015 study by Khanna *et al* that reported patients had some concerns about their wellbeing during a gout attack, as well as side effects imparted by their gout medication. However, these patients also indicated that their current treatment was adequate. The authors concluded that patient-reported outcomes regarding treatment satisfaction are needed to promote better adherence and drive improved clinical and quality of life outcomes.^19^ In line with findings reported herein, Hirsch *et al* determined in a 2010 study that flare frequency and pain were greater determinants of patient-reported impact of gout on quality of life versus common objective measures, including presence of tophi and joint involvement.^20^

### Implications for practice

These results affirm that, for many patients, gout is a painful and debilitating disease that has a significant impact on multiple social and emotional aspects of their lives. The potential for functional and social impairment observed in gout is similar to that seen in other inflammatory arthritic conditions, including rheumatoid arthritis.^2^ Compounding these potential outcomes are patients’ own low expectations of their treatment and their lack of awareness of the importance of reaching sUA target. As outlined in the 2016 updated EULAR guidelines, gout management can be improved by directing treatment based on systematic and frequent sUA monitoring.^6^ Physicians can help patients understand the importance of reaching target sUA as a means to empower actionable discussion based on a measurable biochemical endpoint. Ultimately, an sUA-guided treatment plan may ameliorate pain, functional impairment, and social burden, and help patients achieve better long-term gout care.
